# Effects of maternal social isolation on adult rodent offspring cognition

**DOI:** 10.1038/s41598-023-34834-0

**Published:** 2023-05-12

**Authors:** Robert J. McDonald, Nancy S. Hong, Jan S. Trow, Chelsea Kaupp, R. J. Balog, London Gokarn, Erin A. Falkenberg, Keiko J. McCreary, Nasrin Soltanpour, Carter Witbeck, Aimee McKenna, Gerlinde A. S. Metz

**Affiliations:** Canadian Centre for Behavioural Neuroscience, University of Lethbridge, 4401 University Dr., Lethbridge, AB T1K 3M4 Canada

**Keywords:** Developmental biology, Neuroscience

## Abstract

Prenatal experiences can influence offspring physiology and behaviour through the lifespan. Various forms of prenatal stress impair adult learning and memory function and can lead to increased occurrence of anxiety and depression. Clinical work suggests that prenatal stress and maternal depression lead to similar outcomes in children and adolescents, however the long-term effects of maternal depression are less established, particularly in well controlled animal models. Social isolation is common in depressed individuals and during the recent COVID-19 pandemic. Accordingly, for this study we were interested in the effects of maternal stress induced via social isolation on adult offspring cognitive functions including spatial, stimulus–response, and emotional learning and memory that are mediated by different networks centered on the hippocampus, dorsal striatum, and amygdala, respectively. Tasks included a discriminative contextual fear conditioning task and cue-place water task. Pregnant dams in the social isolation group were single housed prior to and throughout gestation. Once offspring reached adulthood the male offspring were trained on a contextual fear conditioning task in which rats were trained to associate one of two contexts with an aversive stimulus and the opposing context remained neutral. Afterwards a cue-place water task was performed during which they were required to navigate to both a visible and invisible platform. Fear conditioning results revealed that the adult offspring of socially isolated mothers, but not controls, were impaired in associating a specific context with a fear-inducing stimulus as assessed by conditioned freezing and avoidance. Results from the water task indicate that adult offspring of mothers that were socially isolated showed place learning deficits but not stimulus-response habit learning on the same task. These cognitive impairments, in the offspring of socially isolated dams, occurred in the absence of maternal elevated stress hormone levels, anxiety, or altered mothering. Some evidence suggested that maternal blood-glucose levels were altered particularly during gestation. Our results provide further support for the idea that learning and memory networks, centered on the amygdala and hippocampus are particularly susceptible to the negative impacts of maternal social isolation and these effects can occur without elevated glucocorticoid levels associated with other forms of prenatal stress.

## Introduction

During the prenatal period, mammalian brain development is highly susceptible to external environmental influences^1–3^. Prenatal stress (PNS) is associated with numerous childhood brain and behaviour changes, several of which persist into adulthood. Behavioural changes associated with PNS in humans and animals include learning and memory impairments alongside affective changes such as increased emotionality corresponding with a reduced capacity to cope with stress^1,4–6^. Although it is widely agreed upon that PNS for the most part negatively impacts offspring development, some inconsistencies arise when considering how different types and intensities of stress, as well as how stress occurring at different periods during gestation might affect offspring outcomes^6–8^. Animal studies often employ physical stressors such as restraint, bright lights, and loud noises that predominantly occur during a portion of the gestational period. In contrast, gestational stressors in human studies can occur continually, periodically, or acutely. Examples include exposure to maternal stress or anxiety (continual), daily life stressors or stressful life events (periodic) and natural disasters or death of a loved one (acute)^9^. It appears that in animal studies of the functional impacts of prenatal stress, most inconsistencies occur when behavioural measures of learning, anxiety, fear and particularly fear conditioning are employed. Elevated fear and anxiety behaviours are most frequently associated with PNS in rats^10,11^, yet some studies report no changes, or reductions in fear and anxiety associated behaviours particularly when psychosocial^7^ or acute physical stressors are used^6,12^.

Substantial evidence suggests that excessive stress hormones reaching the fetus through the placental barrier are responsible for the impact PNS has on offspring development^4,13^. Rats exposed to PNS develop fewer glucocorticoid receptors which are important for regulating the stress response. Consequently, these offspring have higher basal corticosterone levels and an overactive hypothalamic–pituitary–adrenal (HPA) axis^1,14^. Not surprisingly, most PNS models gauge effects according solely to elevated stress hormones in the mother without considering the impacts on the offspring of the specific stressor administered. Although this was highly effective for gaining initial understanding of PNS and the underlying mechanisms, by only looking at maternal stress hormone levels we likely only highlighted broad behavioural outcomes of PNS. Therefore, further investigations should acknowledge how specific stressors may impact offspring outcomes, particularly models of maternal stress induced via social isolation linked to depression and/or anxiety in humans. For example, exposure to maternal depression during gestation has been associated with childhood behavioural problems and deficiencies in emotional processing, leading to higher occurrences of anxiety and depression^15–17,18^. However, the long-term consequences on adult brain and behaviour are not as evident^14,19^. Often consequences of exposure to prenatal stressors, maternal depression, and maternal anxiety are presented interchangeably, however each specific prenatal stressor may differentially affect offspring. For example, important distinctions in startle reactivity occur in patients with depression and anxiety^20,21^. Depression is associated with decreased fear potentiated startle and diminished affect, whereas anxiety is associated with the reverse. As well, during a discriminative Pavlovian conditioning task, children exposed to maternal depression showed depressed responsivity to conditioned stimuli when compared to children exposed to maternal anxiety^22^.

The stress incurred from socially isolating rats raises circulating corticosterone levels and can be applied continually over a long period^23,24^. There is a small body of work assessing the effects of social isolation on the brain and behaviour. Briefly, this work shows that extended social isolation in young adult rodents can lead to negative outcomes including: dysfunction of hippocampal plasticity mechanisms and associated spatial learning and memory impairments^25,26^; depression-like behaviours^25^; enhanced plasticity and context learning mediated by neural circuits implicated in reward learning and addictive behaviours^27,28^; impaired social recognition^29^; changes in anxiety and attentional processes^30,31^. Surprisingly, there is limited research directed at understanding the effects of maternal social isolation on adult offspring learning and memory functions.

For the present experiment we chose to utilize maternal social isolation stress (MSIS) as a prenatal stressor aimed at modelling the effects of maternal social isolation like that experienced by pregnant women during the COVID-19 pandemic. This work may also, at least partially, provide insights into the impacts of the characteristic social withdrawal seen in patients with major depressive disorder. Long-Evans dams were housed individually prior to and throughout gestation and the behaviour of the dams’ adult male offspring were assessed.

The effects of the social isolation manipulation on the dams’ physiology and behaviour were assessed including stress hormone and blood glucose levels, general locomotor activity, anxiety, and maternal behaviour. A similar profile was performed on the offspring except for the assessment of maternal behaviour.

To assess cognitive function in the offspring, two behavioural tasks were utilized. First, the offspring were trained and tested in a discriminative contextual fear conditioning task aimed at clarifying the conflicting data found in different prenatal stress models on fear learning. The second task is a variant of the Morris Water Task, during which rats are trained to navigate to either a visible or invisible platforms to investigate the offspring’s ability to utilize both stimulus–response habit and spatial learning and memory strategies. This version of the water task was also selected to provide every opportunity for the MSIS offspring to demonstrate spatial learning and memory abilities. The initial cued platform days encourages the rats to swim away from the pool wall, move through the apparatus via swimming, and climb onto the platform for escape. This training decreases the likelihood that the impairments on the invisible platform days are due to a lack of experience swimming, thigmotaxis due to anxiety, not knowing an escape platform exists, etc.^2^.

## Results

### Dams: control and MSIS profile

#### Open field activity monitoring

**Figure 1:**
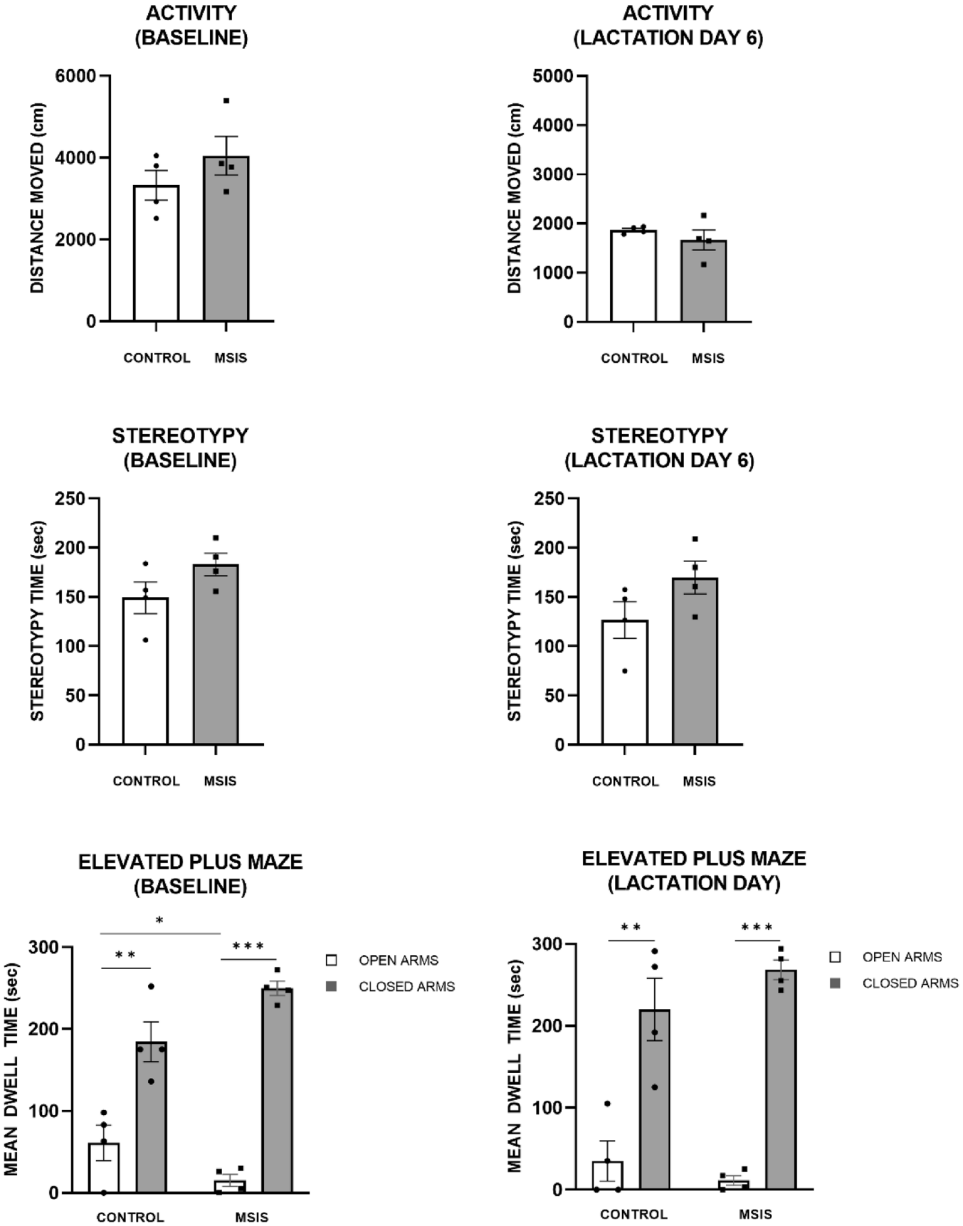
The control (n = 4) and maternal social isolation stress (MSIS (n = 4)) groups were evaluated on their distanced moved (top panel) and stereotypy time (middle panel) in activity boxes during Baseline (left) and at Lactation Day 6 (LD6) testing (right). Both groups had similar activity and exhibited a decrease in activity on LD6 compared to baseline. The amount of stereotypy time was also similar in both groups and did not change significantly between baseline and LD6. The bottom panel shows the results of the elevated plus maze test. At baseline testing, although both groups spent more time in the closed arms compared to open arms (**p < 0.01; ***p < 0.001), the control group spent significantly more time in the open arms than the MSIS group during baseline testing (*p < 0.05). There was no difference between the groups at LD6 testing in the amount of time they spent in the closed and open arms, as both groups had higher dwell times in the closed arms. Data is presented as ± SEM.

The total amount of distance moved, Fig. 1 (top panel), and stereotypy time (middle panel) during a 10 min session was analysed in control and MSIS dams at baseline (left) and LD6 (right). As can be seen, both groups displayed more activity at baseline than at LD6. Univariate ANOVAs on activity indicated no significant effect of group at either testing point [F_(1,6)_ = 1.46, *p =* 0.27, F_(1,6)_ = 0.95, *p =* 0.37]. The same analysis on amount of stereotypy time showed no significant effect of group at either testing point [F_(1,6)_ = 2.983, *p =* 0.14, F_(1,6)_ = 3.01, *p =* 0.13, respectively]. This suggests that the MSIS dams were similar compared to the control dams in both activity and stereotypy time at both baseline and LD6.

#### Elevated plus maze

**Figure 2:**
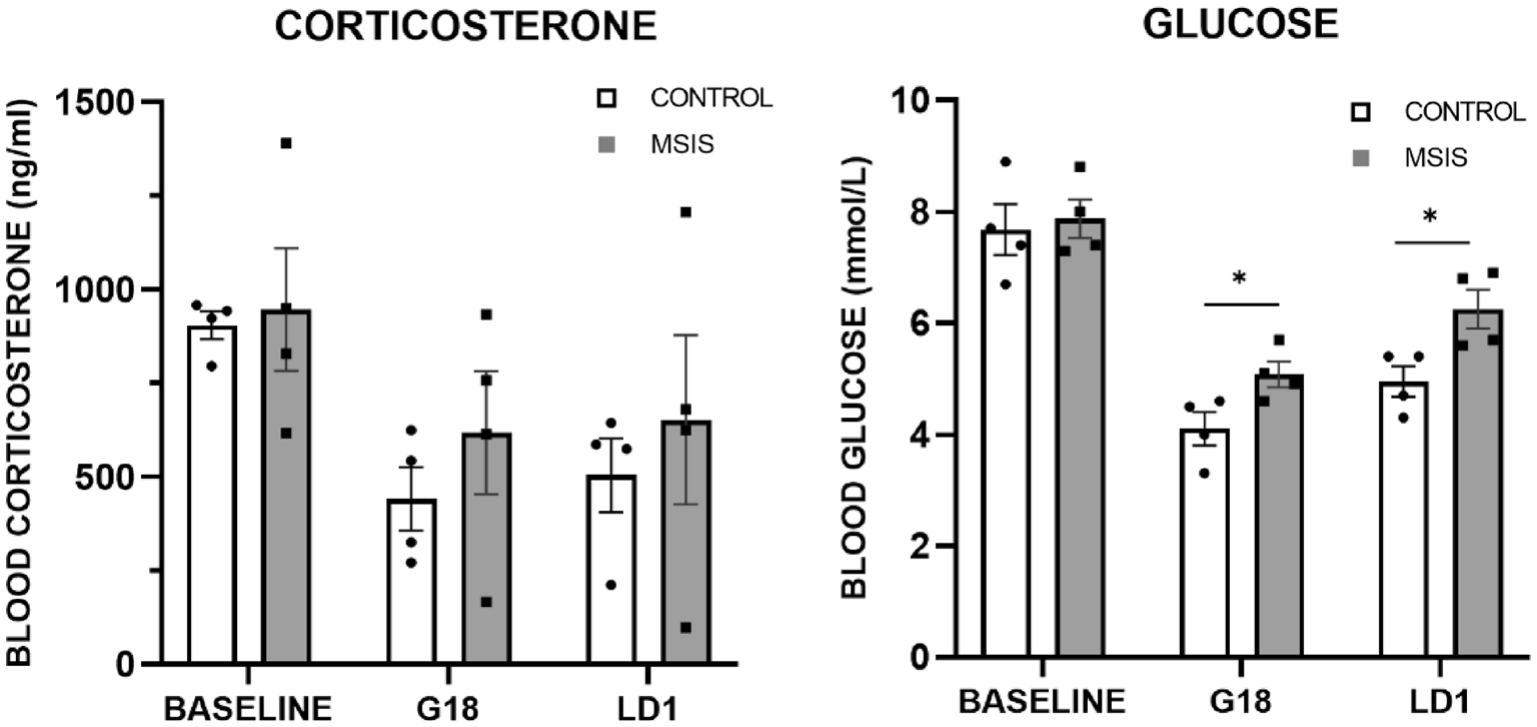
The control (n = 4) and MSIS (n = 4) dams had blood analysed at baseline (prior to pregnancy), G18 (gestational day 18), or LD1 (lactation day 1). (Left) Blood corticosterone levels (ng/ml) did not differ between the control or MSIS dams. There was a significant decrease in corticosterone between baseline to G18 and LD1 for the control (*p* < 0.05), but not MSIS group. (Right) The MSIS dams had significantly higher blood glucose (mmol/L) level than control dams on G18 and LD1 (*p < 0.05). Both groups had a significant decrease in blood glucose levels from baseline to G18 and LD1. Data is presented as ± SEM.

The EPM was used to assess levels of anxiety-like behaviours in the control and MSIS dams. The dams were tested at baseline and at LD6. As illustrated in Fig. 1 (left bottom panel), both groups spent more time in the closed arms compared to open arms at baseline. Upon analysis, a repeated measures ANOVA on dwell time revealed that the MSIS group spent significantly more time in the closed arm compared to the control group [F_(1,6)_ = 6.37, *p* < 0.05]. No group difference was found for the amount of time spent in the open arms [F_(1,6)_ = 4.02, *p =* 0.9]. As can be seen in Fig. 2 (right bottom panel), on LD6 both groups spent more time in the closed arms compared to open arms. No group differences were found in dwell time for closed and open arms (*p*’s > 0.05). The percent of time spent risk assessing was also evaluated and indicated no significant group effect (*p* > 0.05). These results suggest that the MSIS dams may have had increased anxiety-like behaviour as they spent more time in the closed arms at baseline. However, all other measures tested at baseline and LD6 show that the MSIS group are comparable to the controls.

#### Blood corticosterone

Blood corticosterone levels can be seen in Fig. 2 (left) and shows that both groups had lower levels of cortisol at G18 and LD1 as compared to baseline. A repeated measures ANOVA reported a significant effect of blood-corticosterone evaluation day [F_(2,12)_ = 8.47, *p* < 0.006], but no effects of group [F_(1,6)_ = 0.56, *p =* 0.48], nor interaction [F_(1,12)_ = 0.23, *p =* 0.80]. Planned comparisons within the control group revealed a significant difference between baseline and G18 [F_(1,6)_ = 10.76, *p* < 0.02], and baseline and LD1[F_(1,6)_ = 7.46 *p* < 0.04]. Within planned comparisons for the MSIS group did not indicate any significant differences between evaluation days (*p*’s > 0.05).

#### Blood glucose

Blood glucose was measured using a blood-glucose monitor, and the results are displayed in Fig. 2 (right). As clearly seen, there was a difference in blood glucose across days (baseline, G18, and LD1), with a decrease in blood glucose at G18 and LD1 compared to baseline in both groups. A repeated measures ANOVA confirmed these impressions as a significant effect of blood glucose evaluation day was found [F_(2,12)_ = 73.46, *p* < 0.000] and a marginally significant group effect [F_(1,6)_ = 5.40, *p* < 0.059], but no interaction [F_(2,12)_ = 2.21, *p =* 0.15]. To further assess the trending group significance, post-hoc planned comparisons were performed that revealed a significant group difference on G18 [F_(1,6)_ = 6.68, *p* < 0.04] and LD1 [F_(1,6)_ = 8.67, *p* < 0.03]. The results suggest that the MSIS group had higher blood glucose levels than controls prior to and after giving birth.

#### Maternal care

**Figure 3:**
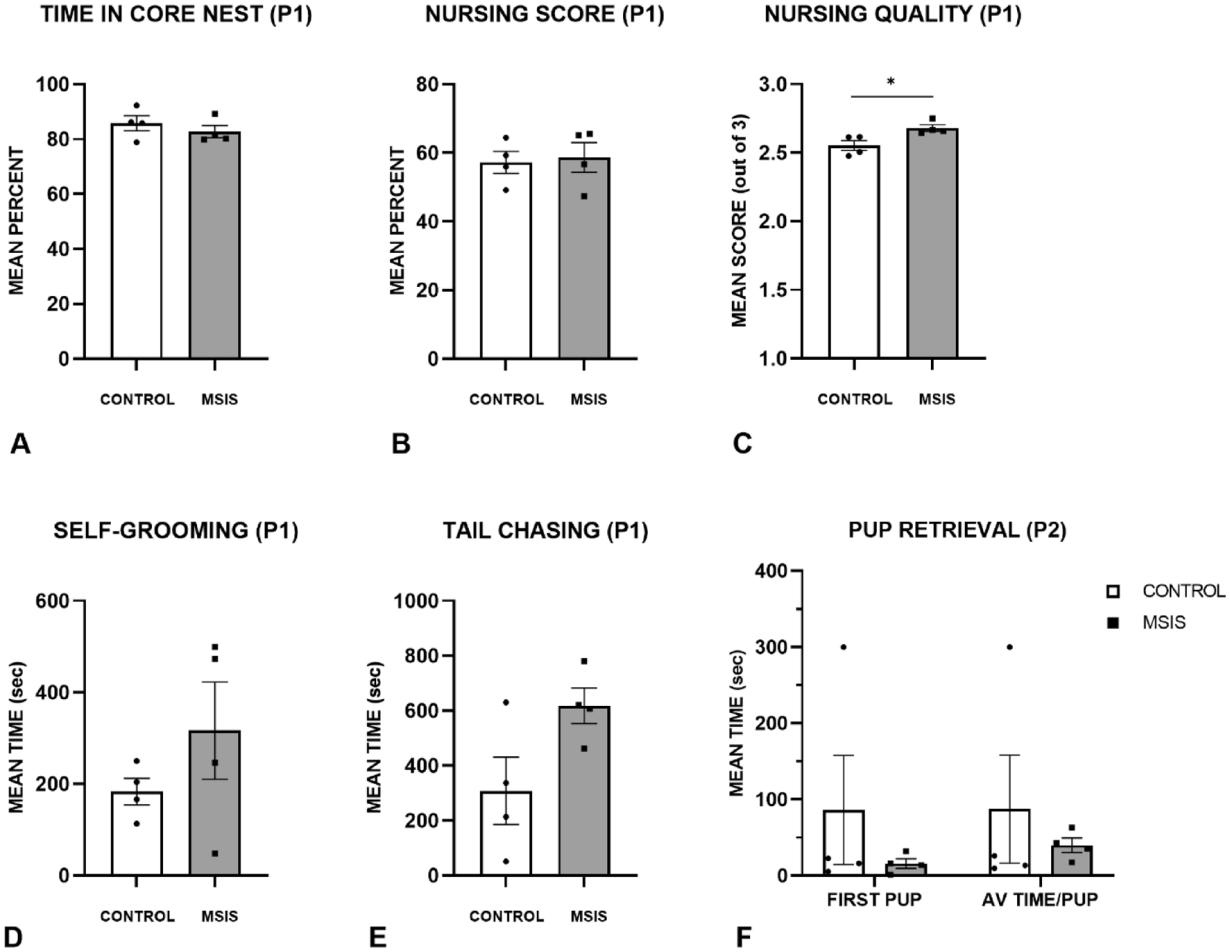
Maternal care and behaviour were scored in control (n = 4) and MSIS (n = 4) dams during the first 10 min of every hour over a 24-h period on post-natal day 1 (P1). Data is presented as ± SEM. (**A**) The percent time spent in the core nest was calculated as total time in the nest divided by total assessment time and multiplied by 100. (**B**) The nursing score percentage consisted of summing the scores (0–3) for each 10-min bin and dividing it by 24 bins. This average was then divided by 30 and multiplied by 100. No difference was observed between the control and MSIS groups in the amount of time they spent in the core nest or their nursing score. (**C**) A qualitative nursing score was also evaluated using the same nursing score except only the scores from 1 to 3 were used to represent the quality of the nursing given. The results revealed that the MSIS dams displayed significantly better nursing than the control dams (*p < 0.05). Other measures scored were the amount of time spent (**D**) self-grooming and (**E**) tail-chasing. No group differences were found on these measures. (**F**) A pup retrieval test was performed on P2 as an indication of the dam’s responsiveness to her pups. No significant differences were found between the groups. The higher mean time displayed by the control group resulted from a dam not retrieving any pups during the 5-min test.

Figure 3 represents the maternal care results for the control and MSIS groups at P1. (A) shows the time spent in the core nest for the control and MSIS groups. As can be seen, both groups spent similar amounts of time in the core nest and a univariate ANOVA confirmed this observation [F_(1,6)_ = 0.75, *p =* 0.42]. The results of the nursing score percentage (B) and nursing quality (C) are also displayed. Although both groups of dams had similar nursing score percentages [F_(1,6)_ = 0.07, *p =* 0.79], the quality of their nursing was different as the MSIS group showed a higher quality of nursing when they were engaged in it [F_(1,6)_ = 8.38, *p =* 0.03]. Similar amounts of time were also seen in self-grooming (D), and tail chasing (E). As displayed in these graphs, and verified with fixed effects ANOVAs, no group difference was seen in self-grooming [F_(1,6)_ = 1.47, *p =* 0.27], but a trend towards group differences was obtained on tail chasing [F_(1,6)_ = 5.02, *p =* 0.066]. To further assess maternal care of the dams, a pup retrieval test was performed at P2. (F) shows that both groups of dams had similar retrieval times and this was confirmed [F_(1,6)_ = 1.14, *p =* 0.33].

**Figure 4:**
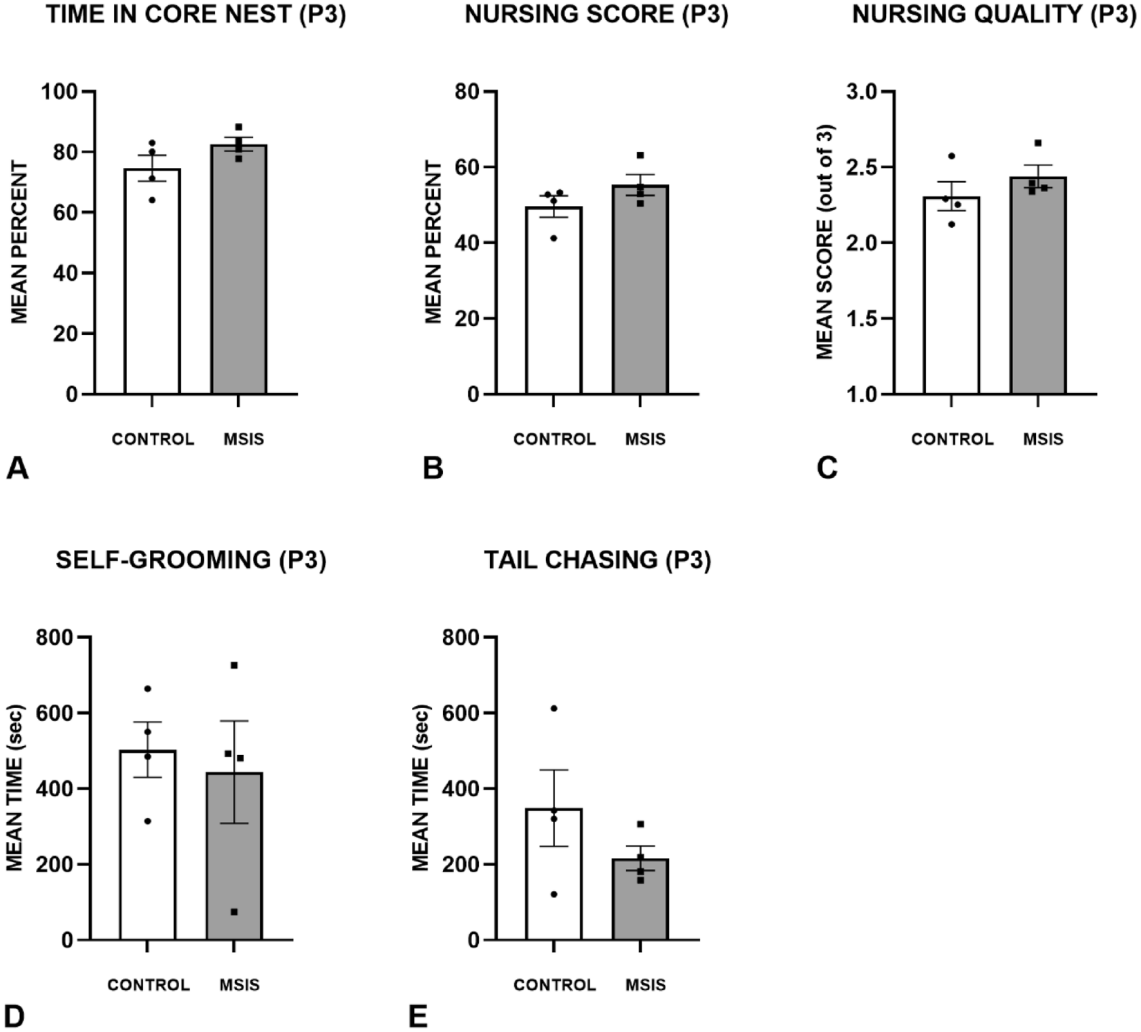
Maternal care and behaviour were assessed in control (n = 4) and MSIS (n = 4) dams during the first 10 min of every hour over a 24-h period on post-natal day 3 (P3). Data is presented as ± SEM. The measures evaluated were (**A**) Percent time spent in core nest, (**B**) Nursing score percentage, (**C**) Qualitative nursing score, (**D**) Self-grooming, and (**E**) Tail-chasing. There were no differences between the groups on any measure of maternal care nor other behaviours scored.

Figure 4 shows the maternal care results for the control and MSIS groups at P3. The percent time in core nest (A), nursing percent (B) and quality (C) for the control and MSIS groups. The groups spent similar amounts of time in the core nest [F_(1,6)_ = 2.68, *p =* 0.15], nursing [F_(1,6)_ = 2.09, *p =* 0.19], and the quality of their nursing was also comparable [F_(1,6)_ = 1.14, *p =* 0.33]. The (D) self-grooming, and (E) tail-chasing also showed no group differences [F_(1,6)_ = 0.15, *p =* 0.71; F_(1,6)_ = 1.57, *p =* 0.26, respectively].

There were some differences in maternal behaviour from P1 to P3, but these were not in the direction one might predict; notably, the MSIS group’s quality of nursing was higher than the control group on P1, although this difference was not seen at P3. Moreover, the control group spent less time in the core nest from P1 to P3, and the MSIS group exhibited a significant decrease in tail-chasing from P1 to P3.

Another measure used to indicate maternal care and responsiveness to her pups is a pup retrieval test. This was conducted on P2, and the results can be seen in Fig. 4E. No group differences were seen in the time it took to retrieve the first pup or the average time spent retrieving each pup [F_(1,6)_ = 0.96, *p =* 0.34; F_(1,6)_ = 1.42, *p =* 0.33, respectively]. The increased variability in the control group was due to one dam not retrieving any pups during the test.

Overall, the maternal behaviour results suggest that social isolation did not negatively impact maternal behaviour indicating that this is not the driver of the learning and memory deficits seen in the offspring of socially isolated pregnant dams.

## Offspring: control-o and MSIS-O profile

### Litter size and offspring subjects

The average litter size was 15 pups for the control group and 13.75 for the MSIS group and this was not statistically different. For the distribution of offspring from the control group, 2 males came from each of 2 dams and 4 males came from each of the other 2 dams for a total of 12 offspring. The MSIS had the same distribution of the males as the controls. The number of subjects assigned to each group was 12.

### Open field activity monitoring

**Figure 5:**
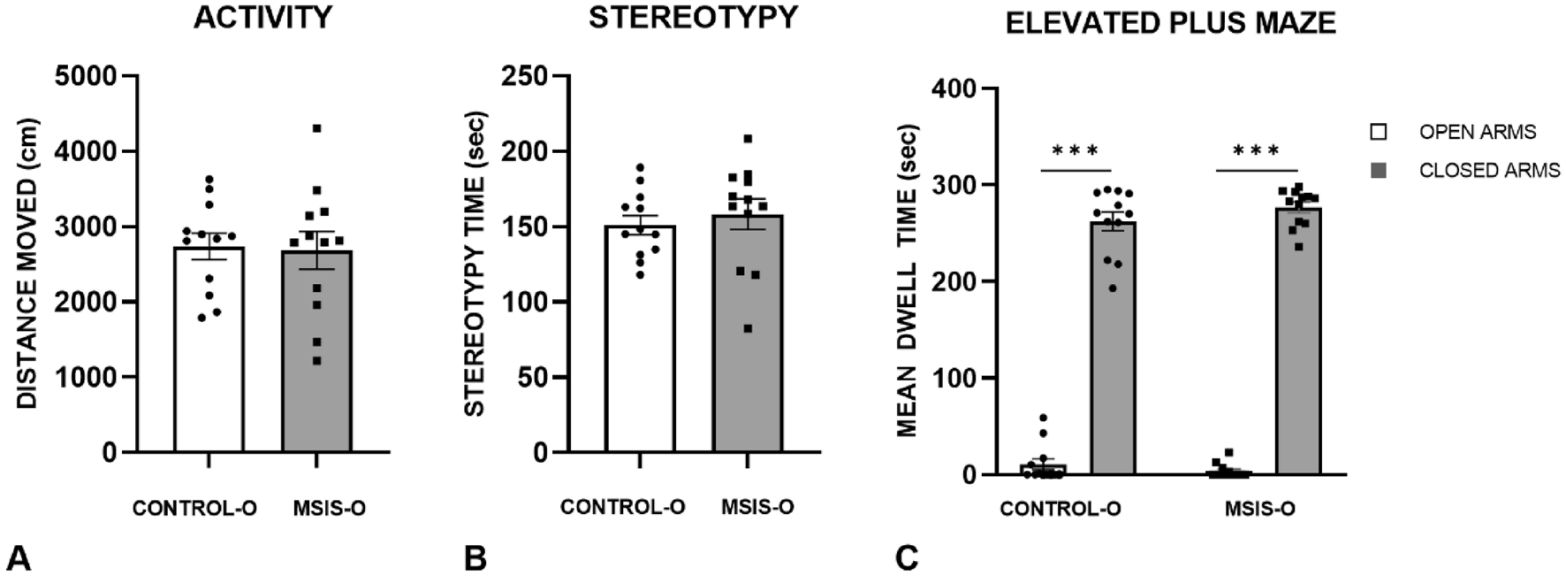
The offspring of the control (control-O (n = 12)) and maternal social isolation stress (MSIS-O (n = 12)) groups were evaluated on their (**A**) distanced moved, and (**B**) stereotypy time in activity boxes. Both groups had similar activity and stereotypy time. (**C**) Shows the results of the elevated plus maze test. Both groups spent more time in the closed arms compared to open arms (***p < 0.001). Data is presented as ± SEM.

Figure 5 represents the total activity (top panel) and stereotypy (middle panel) measures assessed during activity box testing for the offspring of the control (control-O) and MSIS (MSIS-O) groups. A two-way ANOVA with fixed effects performed on the control-O and MSIS-O rats indicated no significant group effects on either of these measures [F_(1,22)_ = 0.03, *p =* 0.87; F_(1,22)_ = 0.37, *p =* 0.55, respectively]. The results clearly indicate no changes in general activity or stereotypy in the adult offspring of socially isolated rat dams.

### Elevated plus maze

The amount of time spent in the open and closed arms are displayed in Fig. 5 (bottom panel). As evident in this figure, both groups spent more time in the closed compared to open arms. Univariate ANOVAs performed on the control-O and MSIS-O rats indicated no significant group effect as both groups spent similar amounts of time in the open and closed arms [F_(1,22)_ = 2.37, *p =* 0.14; F_(1,22)_ = 1.67, *p =* 0.21, respectively]. Similarly, there was no group effect on the amount of time spent risk assessing F_(1,22)_ = 1.67, *p =* 0.21, respectively]. This pattern of results show that adult offspring of socially isolated rat dams do not show increases in anxiety as measured by the elevated plus maze.

### Blood corticosterone and glucose levels

**Figure 6:**
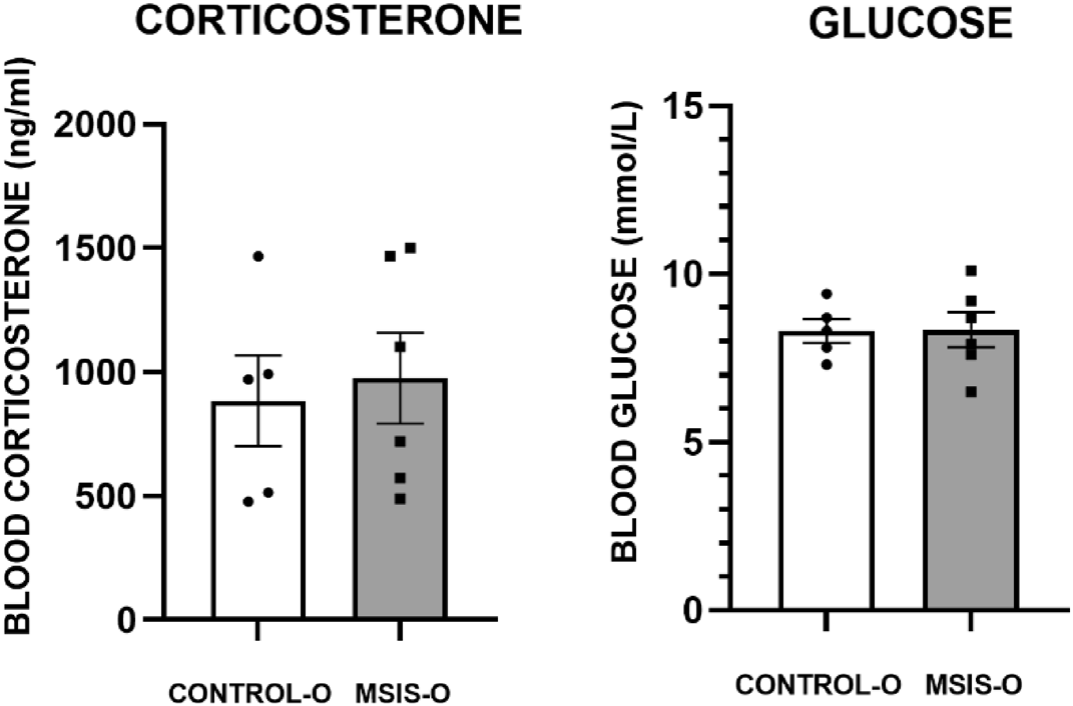
The control-O (n = 5) and MSIS-O (n = 6) had blood extracted for analysis. The results presented here represent the female littermates of the males used in the current study. (left) Blood corticosterone levels (ng/ml) did not differ between the control-O or MSIS-O groups. (right) Blood glucose levels (mmol/L) also did not differ between the groups. Data is presented as ± SEM.

Figure 6 shows the results of the blood corticosterone (left) and blood glucose (right) assessments of the female littermates of the males tested in this study, and as can be clearly seen, there were no differences between the control-O or MSIS-O on either of these measures [F_(1,9)_ = 0.003, *p =* 0.92; F_(1,9)_ = 0.123, *p =* 0.73, respectively]. These results suggest that baseline circulating corticosterone and blood glucose were not altered in the adult offspring of socially isolated rat dams.

### Discriminative fear conditioning to context

**Figure 7:**
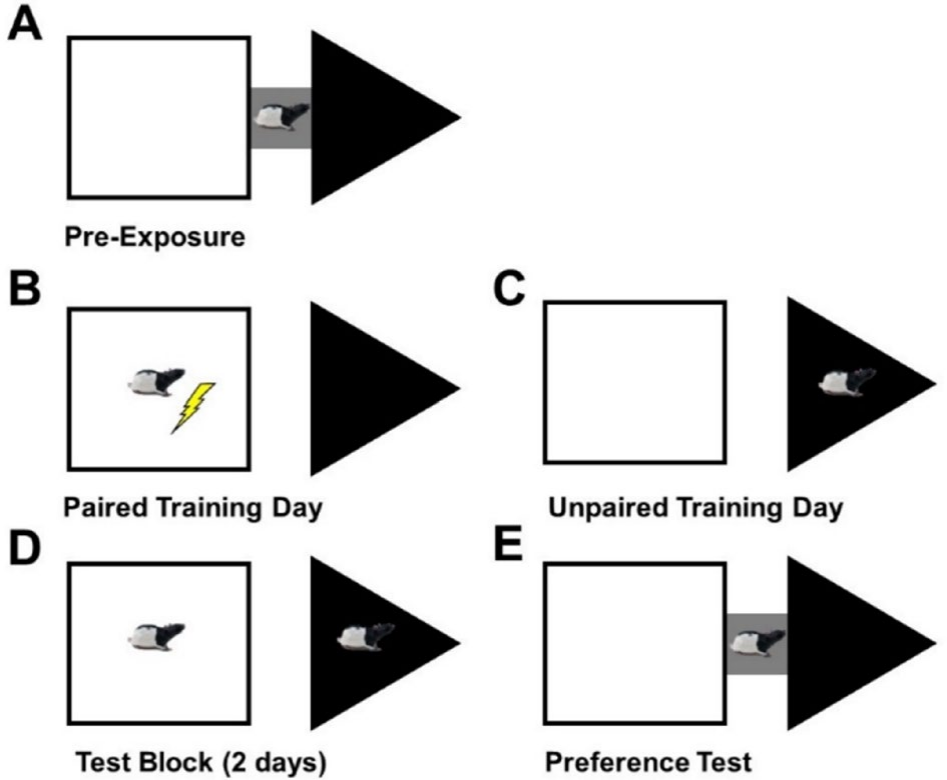
A depiction of the different phases of the discriminative fear conditioning to context task. (**A**) Pre-Exposure. The two contexts differed on multiple dimensions including shape, brightness, and smell. The chambers were connected by an alley which allowed the animals to freely explore both contexts for 10 min during pre-exposure. (**B**) Paired Training. Foot-shocks were administered within the paired context. (**C**) Unpaired training. No foot-shocks were presented in the unpaired context. The paired and unpaired context was counterbalanced. Over 8 training days, rats were exposed to the paired and unpaired contexts on alternating days. (**D**) Freezing Testing. Two assessment sessions occurred, one in the paired and the other in the unpaired context during which time spent freezing was recorded. (**E**) Preference Testing. The connecting alley was opened which allowed the subjects to freely move between contexts [Reprinted from Trow JE, Jones AM, McDonald RJ. (2019) Comparison of the effects of repeated exposures to predictable or unpredictable stress on the behavioural expression of fear in a discriminative fear conditioning to context task. Physiol Behav. 208, 112556].

Data from a subset of the second cohort of rats was removed as it was apparent that the rats received no foot-shocks within the white square due to a malfunction in the wiring. Consistent with this hypothesis, freezing scores for the rats who experienced the white square as their paired context were much lower than what should be expected in both the control-O and MSIS-O group compared to rats in previous cohorts and those that were shocked in the black triangle. Accordingly, the following discriminative fear conditioning to context results are based on 9 control-O and 9 MSIS-O rats. Figure 7 illustrates the apparatus and training paradigm.

#### Pre-exposure

**Figure 8:**
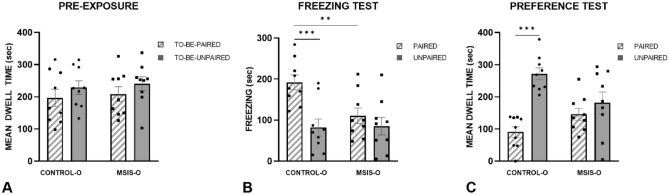
The control-O (n = 9) and MSIS-O (n = 9) were trained on the discriminative fear conditioning to context task. Data is presented as ± SEM. (**A**) Pre-exposure to the contexts indicated no initial difference in the dwell time in each context. (**B**) Following 8 days of fear conditioning the rats were given tests in which the amount of time spent freezing was scored. The results revealed that the control-O group froze more in the paired vs. unpaired context (***p < 0.001), whereas the MSIS-O group did not show this discrimination (*p =* 0.25). The MSIS-O group also displayed less freezing than the control-O group in the paired context (**p < 0.01). (**C**) The preference test showed that the control-O group learnt the discrimination as evidenced by more time spent in the unpaired context vs. paired context (***p < 0.001), but that the MSIS-O group did not display a difference in dwell time between the contexts (*p =* 0.34).

In order to ensure there was no initial chamber preference a two way repeated measures ANOVA was performed on the dwell time accumulated in each context during pre-exposure revealing no significant main effects of group [F_(1,16)_ = 0.94, *p =* 0.35], context [F_(1,16)_ = 1.11, *p =* 0.31], nor interaction [F_(1,16)_ = 0.00, *p =* 0.99]. As can be seen in Fig. 8A, there was no difference between the control-O and MSIS-O groups in the amount of time spent in the to-be-paired and to-be-unpaired chambers.

#### Freezing test

During testing, the amount of time spent freezing within the contexts was used to assess the effect of MSIS on discriminative freezing behaviour. Figure 8B illustrates the groups’ freezing behaviour in the paired and unpaired contexts. Control-O group spent more time freezing within the paired than unpaired chamber; whereas the MSIS-O group did not freeze significantly more in either context indicating that the control-O, but not the MSIS-O rats learned to associate their paired context with foot shock. A repeated measures two way ANOVA on the test block revealed a significant effect of context [F_(1,16)_ = 20.94, *p* < 0.0004], and a group x context interaction [F_(1,16)_ = 8.24, *p* < 0.011], but no significant effect of group [F_(1,16)_ = 2.63, *p =* 0.12]. To elucidate the interaction effect, post-hoc within-group planned comparisons revealed that the control-O group exhibited significantly more freezing behaviour within their paired context than unpaired [F_(1,16)_ = 27.73, *p* < 0.0001], while the MSIS-O rats did not show a significant difference in freezing across contexts [F_(1,16)_ = 1.45, *p =* 0.25]. Furthermore, between group comparisons revealed that the MSIS-O rats exhibited significantly less freezing behaviour within the paired context than control-O rats [F_(1,16)_ = 9.59, *p* < 0.007]. Both groups exhibited similar levels of freezing within the unpaired context [F_(1,16)_ = 0.13, *p =* 0.91].

#### Preference test

Figure 8C represents the overall dwell time of each group in their paired and unpaired contexts. The control-O group demonstrated a strong avoidance of the paired context, however the MSIS-O group did not exhibit a significant preference for either context. A two way repeated measures ANOVA did not reveal a significant effect of Group [F_(1,16)_ = 0.83, *p =* 0.38], but significant effects of Context [F_(1,16)_ = 17.36, *p* < 0.001] and a Group x Context interaction [F_(1,16)_ = 7.74, *p* < 0.02] were obtained. Post-hoc planned comparisons confirmed our observations that the control-o group spent significantly more time in the unpaired vs. paired context [F_(1,16)_ = 24.13, *p* < 0.0001], whereas the MSIS-O group did not show a preference for either context [F_(1,16)_ = 0.96, *p =* 0.34]. Overall, the data suggests that MSIS offspring are impaired in their ability to learn and remember which context was associated with a negative experience. The MSIS-O group also showed lower levels of freezing overall. Moreover, time spent freezing in both contexts was like the time controls spent freezing in the unpaired context. In contrast, control-O rats exhibited significantly more freezing within their paired context indicating they learned to differentiate between the shock and safe chambers.

Taken together, the discriminative fear conditioning to context results indicate that adult male offspring of socially isolated rat dams was severely impaired indicating that a learning and memory network centered on the amygdala was compromised during prenatal brain development.

### Cue-place water task

**Figure 9:**
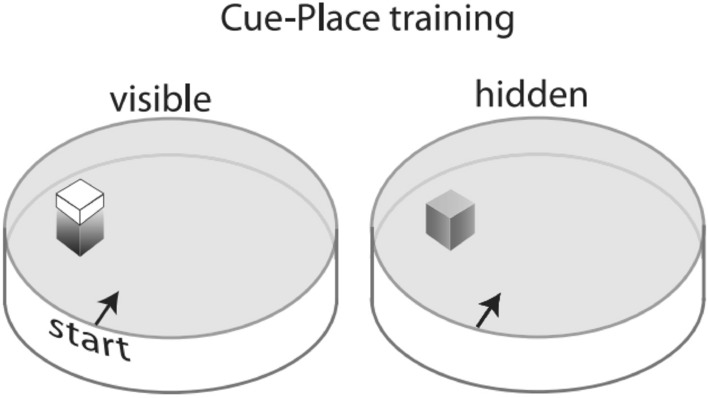
A depiction of the cue-place water task used in the present experiments. Briefly, rats were trained for three days to swim from one of four cardinal start points to a visible platform located in the same place in the pool. On the fourth day, an invisible platform is put in place of the visible platform. This sequence is repeated thrice for a total of 12 training days in which each subject receives nine visible platform and three invisible training days [Reprinted from Gruber AJ, McDonald RJ. (2012) Context, emotion and the strategic pursuit of goals: interactions among multiple brain systems controlling motivated behaviour. Front. Neurosci, 6, 50].

**Figure 10:**
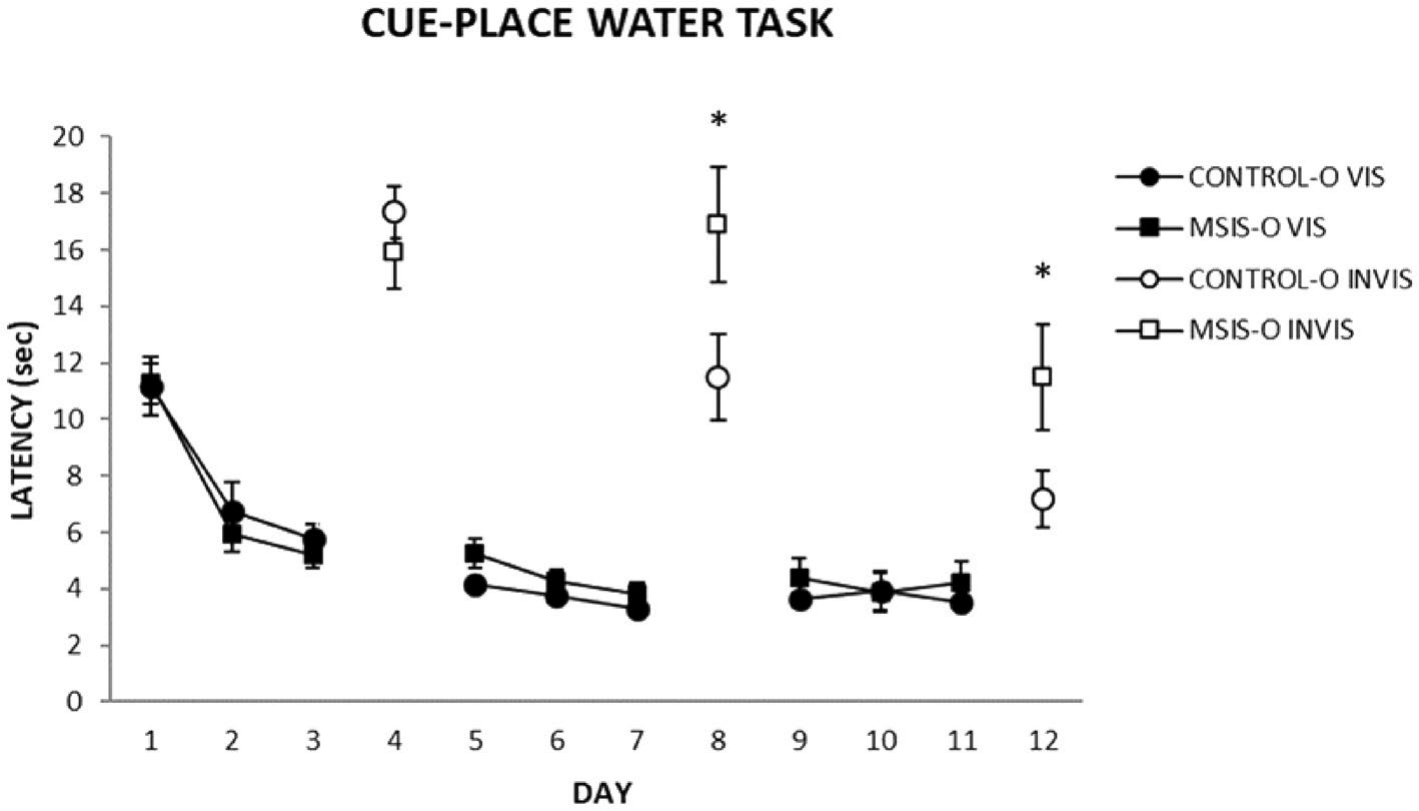
The control-O (n = 12) and MSIS-O (n = 12) groups were trained on the cue-place water task in which they were trained to a visible platform for three days followed by an invisible platform located in the same location, repeated thrice. The closed markers represent the visible training days, and the open markers represent the invisible days. Data is presented as ± SEM. Post-hoc analysis revealed that the control-O group performed better than the MSIS-O group on Day 8 and Day 12 at locating the invisible platform (*p < 0.04, *p < 0.05, respectively). No differences were obtained on the visible platform training days as both groups quickly learned to swim to the platform.

A depiction of the cue-place water task is shown in Fig. 9. Escape latencies were averaged over the 4 daily trials for each rat. As can be seen in Fig. 10, both the control-o and MSIS-O groups quickly learned to swim to the visible platform to escape the pool water. A two-way ANOVA with repeated measures was performed on the daily mean escape latency to find the visible platform (Days 1–3, 5–7, 9–11) and revealed significant main effects of day [F_(8,176)_ = 33.08, *p =* 0.000] and a group x day interaction [F_(8,176)_ = 2.52, *p* < 0.02], but no significant effect of group [F_(1,22)_ = 1.20, *p =* 0.28]. A second repeated measures two-way ANOVA performed on the mean latency to find the invisible platform (Days 4, 8, 12) indicated significant main effects of day [F_(2,44)_ = 18.14, *p =* 0.000], and a group x day interaction [F_(2,44)_ = 4.71, *p =* 0.02], but no significant effect of group [F_(1,22)_ = 3.17, *p =* 0.088]. To further analyze the interaction effect, pairwise comparisons were performed which revealed that the latency for the MSIS-O group to reach the invisible platform on day 8 [F_(1,22)_ = 4.58, *p =* 0.04], and day 12 [F_(1,22)_ = 4.39, *p =* 0.047] was significantly longer than that of the control-O group.

Overall, the pattern of data from the water task indicates that spatial learning and memory processes were compromised in the adult male offspring of socially isolated rat dams while stimulus–response habit learning remained unscathed suggesting that a learning and memory network centered on the hippocampus was compromised during prenatal brain development, but a learning and memory network centered on the dorsolateral striatum was not.

## Materials and method

### Subjects and stress procedure

All experiments were carried out in accordance with the Canadian Council on Animal Care, were approved by the University of Lethbridge Animal Care Committee and are in compliance with the ARRIVE guidelines. Rats were housed in standard polycarbonate shoebox cages (45.5 × 25.5 × 20 cm) on corn cob bedding with food and water available ad libitum. The housing room was kept at a temperature of 20 °C on a 12:12 light dark cycle. Maternal weight gain and litter size were monitored. Prior to reaching adulthood dams were housed in a cage of three and separated into groups. In general, all the procedures were done in young adults. On postnatal day 90 (P90), 4 females to be included in the treatment group were removed from their home cages and singly housed in new cages, these females constituted the P90 maternal social isolation stress group [(MSIS group)^32^. P90 was selected to start isolation stress so that the females were young adults which should be a formative period to assess lifelong effects of adult stress.

### Breeding procedure and body weight

Breeding began on P110 and ensured that dams had preconceptional isolation stress for a minimum of 20 days. This way, the stress was salient before and during pregnancy for all MSIS rats. The first successful breeding occurred on P110 and the latest occurred on P158. The need for multiple mating sessions happened relatively equal in the experimental and control groups. Breeding was done by placing a male and female together for 30 min each day. Following each breeding session, the control females were placed back in their home cage pair housed, and the MSIS females were placed in their home cage singly. This was repeated until the females got pregnant. This breeding procedure ensured that the MSIS females remained socially isolated except for the breeding sessions. For breeding, 8 males were used with 6 males being successful, and each dam had one litter. Upon conception MSIS animals remained isolated. The 4 control females were housed in pairs throughout gestation and only isolated a day before parturition to ensure a safe and healthy birth. Pups remained with their mothers until postnatal day 21, at which time they were weaned, and pair housed. Once the pups reached P90 the male offspring commenced behavioural testing. These specific postnatal days were selected based on previous work from our labs and others^7,32–35^.

We measured the dams’ body weight before experimental procedures and then 2 days before birth (G21). The average body weight of the controls at baseline was 316.4 g and 333.9 g for the MSIS group. At G21, the control group had gained 145.7 g and the MSIS group gained 148.6 g from their baselines. None of these group differences were statistically different.

### Open field activity monitoring

Locomotor activity was recorded using Accuscan activity monitoring system, comprised of clear Plexiglas boxes (L 42 cm, W 42 cm, H 30 cm) connected to a computer. The rats were placed in the activity boxes and activity was recorded for 10 min. The rats were returned to their home cages after the testing session. For the control and MSIS dams, locomotor activity was assessed prior to pregnancy on P103 (baseline) and at LD6. The control offspring (control-O) and MSIS offspring (MSIS-O) locomotor activity was assessed at P90.

Locomotor activity recorded on a computer with VersaMax program was converted to spreadsheets using VersaDat software (AccuScan Instruments, Inc.). The measures of activity assessed were total horizontal activity and total stereotypy.

### Elevated plus maze

To measure anxiety-like behaviours, the rats were tested on the elevated plus maze (EPM) for 5 min. The apparatus was constructed from black Plexiglas consisting of two open and two closed arms (each 50 cm × 10 cm) that was elevated 100 cm above the floor. The open arms had no side or end walls, and the closed arms had side and end walls (40 cm high). The experimenter placed the rat in the center of the plus maze facing an open arm for 5 min. The session was recorded, and a blind observer later scored the amount of time in closed and open arms, as well as a percentage of time risk assessing for each subject. A rat was determined to be in the closed or open arm when its front paws and upper body were in the arm. Risk assessment was determined to be the total amount of time at the edge of a closed arm with its nose towards the center platform. The control and MSIS groups were tested on the EPM prior to pregnancy on P104 (baseline) and LD6. The control-O and MSIS-O groups were tested on the EPM at P94.

### Blood corticosterone and glucose analysis

Blood samples for corticosterone analysis were collected from the animal’s tail vein while they were under isoflurane anaesthesia for approximately 5 min. 0.2 ml of blood was collected from each rat using a 23ga butterfly needle attached to a 1 ml syringe that was then transferred to an Eppendorf tube and immediately placed on ice. The blood samples were centrifuged at 4 °C at 3000G for 10 min. The plasma was extracted and stored at – 80 °C until running a Cortisol ELISA Kit (ab108665). To assess blood glucose levels, a small amount of blood was obtained from a tail poke, and then measured using a One Touch™ glucose monitoring system (Bayer). All dams underwent blood sampling on P105, gestational day 18 (G18) and lactation day 1 (LD1). P105 was chosen to get a measure of blood cortisol and glucose following 2 weeks of social isolation in the MSIS group. G18 was chosen to obtain blood cortisol and glucose levels a few days prior to parturition, and LD1 as it is the first day of lactation. Although male offspring of the control and MSIS groups in the present study did not undergo blood sampling, their female littermate cohorts did have their blood corticosterone and blood glucose levels assessed and their results are presented in the present paper. This occurred because our collaborators who were studying transgenerational stress never planned on testing this first generation of subjects. They took the females’ blood at P105 as they were interested in this data because these females were going to be the next generations mothers. The original plan was that the males were not to be used for anything after this point.

### Maternal care

Video recordings were made of each dam in their home cage from G18 to post-natal day 7 (P7). An experimenter blind to the dam’s condition scored each dam on day P1 (24-h after giving birth) and P3 (72-h after giving birth) for 10-min every hour for a 24-h period. Each bin was scored for time in the core nest, nursing, self-grooming, and tail chasing. Time in core nest and nursing were measures taken as indications of maternal care to pups.

*Time in core nest* was accrued when the dam entered the location of her pups and covered most of the pups with her body. Time would stop when the dam would leave the boundaries of the primary nest area. If the dam was resting, eating, or drinking on the edge of the nest but was not entirely in the nest, she was not considered as being in the nest.

*Nursing* was broken down into nursing score and nursing quality. Nursing score consisted of a 4-point system that was used to determine the type of nursing exhibited by the dams at each minute mark during the 10-min bins and were considered a snapshot. Depending on the positions of the dam, at the point of the snapshot, a score out of three was given that represented the amount of effort and engagement of the nursing displayed. A score of zero was given if the dam was located in the nest but was not participating in nursing, e.g., grooming, eating, and drinking, or if she was outside the nest. A score of one was recorded for a dam lying directly on her side with the pups feeding with no part of the dam covering the litter of pups. A score of two was given if the dam was over the top of the litter and lying on top of the pups. The highest score of three was recorded when the dam was over the top of her pups with an arched back and extended limbs. The Nursing Score percentage consisted of summing the scores for each 10-min bin and dividing it by 24 bins. This average was then divided by 30 and multiplied by 100. The nursing quality used the same Nursing Score snapshots except only the scores from categories 1–3 were used to represent the quality of the nursing given. Therefore, the quality score only reflected the snapshots when the dam was engaged in nursing.

*Self-grooming* was measured as an indication of maternal care as we surmised that a dam that was grooming herself would also be able to care for her pups. This measure was scored only when the dam was seen grooming herself in the close vicinity to the core nest because at certain angles it was difficult to distinguish whether the dam was grooming herself or her pups. Self-grooming was defined as licking, biting, and scratching of her body, as well as using her paws to clean her head, ears, and facial area.

*Tail chasing* time started when the dam noticed and was focused on her tail, initiating the start of the spin. Once the dam began this action, the time was recorded until she ceased active turning to chase it or dropped the tail from her mouth and disengaged with it^35^. This measure may serve as an indicator of maternal stress and anxiety-like behaviours^35^.

*Pup retrieval* was tested as a measure of maternal care and responsiveness of the dams. This formal test occurred on P2 and consisted of removing the dam from her home cage and manually placing her pups in various locations throughout the home cage but outside the core nest. The dam was then returned to the home-cage and the time to return all displaced pups to the core nest area was recorded^36^.

### Discriminative fear conditioning to context

The control-O and MSIS-O groups were tested on the discriminative fear conditioning to context (DFCTC) paradigm to assess amygdala function at P110. A pictorial representation of the apparatus and method can be found in Fig. 9. Behavioural tests were performed with lights on in the testing rooms. The behavioural testing occurred during their light phase (light/dark housing schedule) so that their circadian rhythms would not be disrupted coming from the dark into the light for behavioural testing.

#### Apparatus

Two context chambers were used that differed on three dimensions: colour, shape, and odour. One context was a white square-shaped chamber measuring 41 × 41 cm at the base and a depth of 20 cm; the other context was a black triangle-shaped chamber measuring 61 × 61 cm at the base with a depth of 30 cm. Both context chambers had a floor made of stainless-steel bars spaced 1.5 cm apart. A small plastic cylinder containing a distinct odorant was mounted on one wall of each chamber. Daily, a drop of each odorant, serving as an olfactory cue, was placed on a cotton ball that was inserted into the chamber. Iso-amyl-acetate served as the olfactory cue with the black triangle chamber, and eucalyptus served as the olfactory cue with the white square chamber. During pre-exposure and preference testing, the two chambers were connected by a grey alley (16.5 cm long × 11 cm wide × 11 cm high). The entire structure was placed on a clear Plexiglas table with a height of 100 cm. A mirror (91 cm long × 61 cm wide), inclined by 45°, was placed on the floor under the clear table, allowing the experimenter to see the interior of the chambers. A video camera was placed in front of the mirror to record the testing and preference phases of the experiment. The entire apparatus was cleaned with a soap solution after each rat.

#### Pre-exposure

Rats were placed in the middle alley and allowed to freely explore both chambers for 10 min. Dwell time was accumulated when both forepaws and half the body were past the threshold of the doorway into one of the chambers and ended when both forepaws and half the body were past the threshold of the doorway into the alley. According to pre-exposure scores the rats were counterbalanced such that any innate preferences for either context was balanced. For each group, half the rats experienced their paired context as the white square and the other half were paired in the black triangle. These rats were further subdivided so that half the rats from each group experienced their paired context on the odd number days (1, 3, 5, and 7) and the unpaired on the opposing days (2, 4, 6, and 8). The other half of the rats experienced the paired context on the even number days of training (2, 4, 6, and 8) and experienced their unpaired context on the other days (1, 3, 5, and 7).

#### Training

Training began approximately 24 h following pre-exposure. All paired days occurred in room A, and all unpaired days occurred in room B. During training, Plexiglas panels were inserted to block access to the middle alley. In the unpaired condition, each animal was placed in its assigned context individually and remained there for 5 min. For the paired (foot shock) condition, 0.6 mA of current (scrambled shock) was delivered through the grid flooring at min 2, 3, and 4. This training sequence was repeated four times for a total of eight training days. Rats alternated between experiencing their paired (foot-shock) context and their unpaired (neutral) context on opposing days.

#### Testing blocks

Testing was conducted to determine if the groups learned which chamber predicted the aversive event. Testing began approximately 24 h following the final training session. No shocks were administered during the tests and all testing sessions occurred in room B. On each of the testing days, rats were placed within the apparatus for 5 min and the session was recorded. Time spent freezing was scored by an observer blind to the experimental conditions. Freezing constituted total immobility of the rat’s body and whiskers, other than the movement required for breathing. The testing block consisted of two consecutive test days during which all the rats spent one of the days confined within their paired context, and the other day confined within their unpaired context.

#### Preference test

Preference testing determined if the rats show an aversion to the context previously paired with shock. Preference occurred approximately 24 h following the final day of testing. The grey alley was replaced so that the two contexts were connected. Rats were placed within the middle alleyway and allowed to freely explore both chambers for 10 min. Dwell time in each context was recorded by a blind observer. Time was accumulated when both forepaws and half the body were past the threshold of the doorway into one of the chambers and ended when both forepaws and half the body exited the chamber into the alleyway. The preference test occurred in room B.

### Cue-place Morris water task (MWT)

The control-O and MSIS-O groups were tested on the cue-place version of the MWT at P130. This task assesses both dorsolateral striatum (cue-visible platform) and hippocampal (place-invisible platform) network functions.

#### Apparatus

A white plastic pool 154 cm in diameter and 50 cm in height was filled with water (20-21 °C) to a level of 31 cm and made opaque by adding non-toxic white paint (Tempera). A Plexiglas platform 28 cm tall, with a 12 cm × 12 cm top was submerged 2–3 cm under water. The training room was 310 cm × 610 cm, and the pool was raised 48 cm above ground in the center of the room. All extra maze cues remained unchanged throughout all trials including posters of varying size, a computer desk, chair, the experimenter, a sink cabinet, and door. A computer tracking system (Ethovision, Noldus, Leesburg, USA) was used to collect data obtained from an overhead video camera. The tracking system recorded latency and path length to reach the platform, as well as quadrant preference.

#### Procedure

The animals were trained for 12 consecutive days. A 3:1 training schedule was repeated three times over the 12 days. The platform was placed in the same quadrant for all 12 days of training. For the first three days of training the platform was visible, but on the fourth day an invisible platform replaced the previously visible platform, remaining in the same location. This was repeated three times so that animals received a total of 9 days training with the visible, and 3 days with the invisible platform. During training, each subject received 4 trials per day with 4 pre-determined start points. The circular pool was conceptually divided into four quadrants of equal size, and the 4 start points were chosen to be at the four intercepts of the two dividing axis and the perimeter of the pool. Animals were run in a distributed manner and were allowed a maximum search time of 30 s in the pool. A trial ended when the subject located the hidden platform, or after 30 s had elapsed, in the latter event the rat was guided to the platform by the experimenter. The subject was required to remain on the platform for 10 s before being returned to its holding cage.

## Discussion

The current study investigated the effects of maternal social isolation (MSIS) on learning and memory functions and fear in adult male offspring. The results showed that maternal social isolation produced functional impairments in offspring including disrupted discriminative fear conditioning to context as measured with conditioned freezing and preference tests. Specifically, the offspring showed dampened levels of fear and impaired learning abilities on this task. Further, these same subjects were impaired at the spatial learning components of a navigation task but not the cued learning components. Taken together, this pattern of results suggests that maternal social isolation impacts learning and memory functions mediated by neural networks centered on the amygdala^40,37^ and hippocampus^38,39^ in offspring that lasts into adulthood.

Overall, these results indicate that social isolation of pregnant dams has considerable long-term effects on male offspring behaviour persisting into adulthood. Most notably, these offspring exhibited depressed and generalized freezing after contextual fear learning. When given free access to both chambers the MSIS group showed no preference for either chamber suggesting a persistent impairment on this context discrimination task. In the cue place water task both groups exhibited similar overall learning curves across days, however the MSIS group were slow to reach the invisible platform on the 8th and 12th day (2nd and 3rd place learning day) when required to use a hippocampal-based spatial navigational strategy^39^. Taken together, this pattern of results shows that general learning abilities are not impacted but learning and memory processes associated with fear conditioning and spatial learning and memory were affected in offspring of mothers that were socially isolated.

### Discriminative fear condition to context

This task measures whether rats have learned to associate a context with the aversive foot-shock received within that context during training. Moreover, rats must also properly discriminate their shock from safe context within which rats receive equal exposure and training, however in absence of shock. Learning is considered to have occurred when fear is exhibited in absence of the shock expressed as elevated freezing within the shock context as well as active avoidance of the shock context during preference. The discriminative nature of this task allows for more sensitive behavioural measures which not only highlight learning deficiencies but also any discrimination deficits. The ability to learn this association and proper expression of freezing and preference requires participation from a neural network centred on the amygdala, whereas the orbital prefrontal cortex is important for discriminative freezing yet plays no role in preference^40,41^. The hippocampus has been implicated in discriminative fear conditioning to context, particularly in the retrograde direction^42^ but extrahippocampal circuits can support this kind of learning as well^43,44^.

Contrary to the predominant assumption that MSIS in rats is associated with elevated anxiety and freezing^10,14^ our results show that maternal social isolation results in depressed freezing with no active avoidance of the shock chamber. This pattern of effects may be best explained by amygdala rooted dysfunction as rats with amygdala damage or reversible inactivations show similar depressed fear and an inability to discriminate the two contexts^40^.

In children and infants, stress that occurs late in gestation has been shown to be a robust predictor of fearful temperament and elevated anxiety^6,45^. Therefore, the lower levels of fear seen in the MSIS subjects could be on account of habituation to the stress that develops throughout gestation. Additionally, milder prenatal stress has been shown to induce a fear of novelty as well as learning deficits, however does not impact offspring HPA axis as a more severe stressor might^19,46^. Finally, these results are very similar to a human study that compared offspring exposed to maternal depression and maternal anxiety in a discriminative Pavlovian conditioning task. In this study, as well as our task, participants were trained to associate a CS + (paired context) with an aversive event alongside training with a CS- (neutral context) that was not associated with the aversive event. Exposure to maternal depression, similar to exposure to maternal social isolation led to depressed skin conductance responses to the CS + when anticipating the aversive event. Yet aside from the indication of some generalization there were no major group differences in reactions to the CS-, which is consistent with our results^22^. Therefore, it seems that the attenuated generalized fear results we report in offspring of rat dams that experienced isolation are different from most other MSIS studies. However, the depressed generalized fear we report is consistent with children of mothers depressed during gestation. This could be due to the type of stress the mother endures during isolation or depression or because of the extended and continual exposure to the stressor.

### Cued and spatial learning in the Morris Water Task

Throughout the task the platform switches from a visible to invisible platform engaging the use of cue and spatial learning respectively. Spatial learning on the invisible days is reliant on the hippocampus, however the visible platform location can be acquired using either spatial or response strategies^39^. Our results indicate that MSIS produces hippocampal-based spatial learning impairments highlighted by the impairments to reach the platform on the last two place days (days 8 and 12). Performance on visible platform days is similar in both groups with uniform learning occurring across days. This is consistent with several previous studies indicating that prenatal stress results in impairments in spatial learning^6,47,48^. Additionally, ample evidence in human and rat studies indicate reduced hippocampal volume in prenatally stressed offspring which could account for the spatial deficit^1,4,13,49^. Both lifetime stress and prenatal stress in humans result in a bias towards the use of cue learning strategies mediated by the dorsolateral striatum^50^. Similarly, rats trained in this cue place task prenatally exposed to alcohol were biased towards the use of cue strategies although they had no spatial acquisition deficits on any invisible platform days^2^. Although a competition task did not occur in this study, it is likely that the spatial impairments would translate into a similar response strategy bias as is seen in these studies.

### Other impacts of social isolation on mothers and offspring

Clearly, social isolation endured by a pregnant rat dam impaired learning and memory networks centered on the hippocampus and amygdala, but what is driving these effects remains to be determined. For this study, we profiled physiological and functional impacts of social isolation in dams and offspring. For the dams, we assessed stress hormone and blood glucose levels before, during, and after pregnancy. We also measured general activity levels, stereotypy, and anxiety using the elevated plus maze. Finally, following birth we tracked different components of mothering behaviour to determine if the social isolation led to impaired interactions between the dams and offspring that could lead to cognitive impairments in adulthood. The results were surprising in that neither the dams nor their offspring showed differences in baseline blood-borne stress hormone levels (cortisol). There were also no differences in locomotor activity or anxiety levels.

One interesting effect we did find was that the MSIS dams did show elevated blood glucose levels at gestational day 18 (GD18) and lactation day 1 (LD1). This provides a potential clue as to a mechanism driving the changes in hippocampal and amygdala function, but further work is required to assess this idea. There is some preliminary evidence that changes in glucose metabolism during gestation under stressful conditions can lead to changes in offspring physiology and brain function^51^. However, it is important to note that when we looked at this measure in the larger cohort of dams the GD18 effect was still there but smaller and the LD1 effect was not reliable. Regardless, it does seem like there are changes in blood glucose levels in isolated dams that appear to emerge during gestation. Further work is required to ensure that this effect is reliable, potentially via experiments in which the subjects are fasting during blood draw or challenged with a dietary manipulation. We will also assess this as a potential mechanism for impairing hippocampal and amygdala learning and memory functions.

Additionally, it is widely accepted that postnatal care has vast impacts on offspring development and prenatal depression has been linked to poor post-natal care in humans^52–54^. Despite this type of evidence, the dams that experienced social isolation, in the present study, showed normal patterns of maternal behaviour suggesting that this is not a driver of the impairments in learning and memory function in their offspring.

### Social isolation stress

Social isolation can occur for a variety of reasons including personality traits, socio-economic status, and predispositions to mood disorders^55^. These factors can have serious consequences if pregnancy is in the mix, although these impacts are not well understood and understudied.

People during societal lockdowns can also experience social isolation stress even when they can see other people in their homes and yards, hear and see them on virtual meeting platforms, but lack the ways of in-person interactions. This kind of isolation can also be exacerbated by low socio-economic status, single-parenthood, and predispositions to mood disorders. Recent findings revealed that mothers are particularly vulnerable to the effects of social isolation^56^. For example, social isolation represented a significant risk factor for elevated depression and anxiety along pregnant mothers during the COVID-19 pandemic^57^. During social isolation rats have similar experiences, they are housed individually so they are unable to interact with other rats although they are still able to hear, smell and see rats in neighbouring cages.

Rats being socially isolated have elevated circulating corticosterone levels, yet to our knowledge there is little published literature in rats utilizing social isolation as a prenatal stressor. Previous studies showed, however, that MSIS during pregnancy causes robust transgenerational phenotypes of adverse birth outcomes^58^, reduced motor skills and motor tract density in the F3 generation^39^, and anxiety-like behaviours and impaired HPA axis regulation in the F3 generation^33^. Most prenatal stress models are primarily gauged on elevated corticosterone in the dams and elevated basal corticosterone levels are characteristic of depressed individuals^59^. Therefore, by socially isolating pregnant dams we can model the psychosocial type of stress incurred during depression, as well as elevate circulating corticosterone levels in the dams^23,24^. As discussed previously and shown in the present study, it seems that isolation stress has different effects on offspring brain function and associated behaviour compared to other forms of prenatal stress, but little is known about the effects of MSIS on offspring brain and behaviour. Furthermore, we did not find that that MSIS produced elevations in stress hormone levels in the dams or offspring suggesting that isolation can activate multiple mechanisms and pathways that can lead to brain dysfunction independent of elevated corticosterone levels^60^.

### Caveats

This study only presents results from male offspring. We are acutely aware of the fact that prenatal stress has been widely reported to impact males and females differently. However, this study exists because we were given access to male rats that were part of a large-scale study assessing the effects of transgenerational stress on the brain and body, and the female offspring from our study were used to generate a new generation of offspring in that ongoing project. The collaborating laboratories wanted to get as much data from the male offspring as possible as this is an explicit goal of our funding and animal care agencies, to reduce the amount of animal subjects used. This study was conceived and executed with this opportunity and goals in mind. Future work from our lab will assess the effects of MSIS on female and male offspring now that we have found the interesting pattern of results reported here. It is important to note that based on previous work, it is likely that the female offspring will show worse functional impacts than males.

Another potential weakness of the present study is that baseline blood corticosterone was not assessed in the male MSIS offspring. The question remains whether the male MSIS offspring showed elevated baseline blood corticosterone levels, but several lines of evidence suggest that the male MSIS offspring did not show alterations in blood corticosterone levels. First, female MSIS offspring from the same study did not show these effects and, if anything, females should be more likely to show the impacts of prenatal stress on elevated corticosterone levels or the development of anxiety and depression^61^. Second, although it is likely that baseline blood corticosterone levels were not altered in the MSIS offspring it is possible that challenges to the stress response could be exaggerated. Although possible it seems unlikely as we have no indication of elevated fear or stress during discriminative fear conditioning which should occur if basal or induced blood corticosterone levels were elevated in the male MSIS-O. Finally, the male MSIS-O showed no increases in anxiety-like behaviour levels, as measured on the elevated plus maze, which would be expected to emerge if basal or induced blood corticosterone levels are elevated^62^.

A third caveat, when interpreting the present results is that it is important to be careful in generalizing the results to less stressful learning situations, like that found in learning appetitively motivated tasks, as we cannot necessarily say that the impairments in male offspring due to MSIS is impacting a rat’s ability to properly learn and remember or if behavioural changes are due to an inability in handling stress leading to learning impairments.

Finally, we were concerned that the number of dams (n = 4/group) was inevitably small due to the number of subjects required for the offspring assessments, and that this may have impacted the statistical power in the analysis of the maternal data resulting in an inability to detect differences on important measures of stress like stress hormone levels in the dams. To alleviate this concern, we obtained maternal data from additional dams that were treated identically to the dams used in the present study to increase the group sizes. These results are reported in Supplemental Figs. 1–4. As can be seen, these results are very similar to the original rat dams in the present study. For example, the only anxiety-like behaviour displayed was during EPM at baseline testing in which the MSIS dams spent less time in the open arms than the control dams. There were no differences between the MSIS and control dams on other measures such as elevation of stress hormone levels, maternal behaviour, and other forms of anxiety, etc.

### Future work

Future work will be directed at questions raised by the results reported in this study. First, replicating the current experiments with both male and female offspring to identify sexual dimorphisms in the cognitive and affective functions. Second, experiments will be designed to ascertain the nature of the brain changes in the neural networks centered on the hippocampus and amygdala and to see if these are different from the effects of prenatal stress in which blood stress hormones are elevated. This will include an assessment in these regions of dendritic changes (spines, branching, etc.), biochemical pathways implicated in plasticity, electrophysiological correlates of learning and memory, and systems dynamics using calcium imaging. Finally, we will assess inflammatory and other stress marker in this maternal isolation stress model^58^.

## Conclusion

This study assessed the effects of a specific form of prenatal stress on adult learning and memory functions associated with brain networks centered on the hippocampus and amygdala. Specifically, we used a maternal social isolation stress procedure to model the stress associated with pregnant women finding themselves socially isolated due to unusual life circumstances or isolation during depressive episodes. The results showed that learning and memory functions dependent on neural networks centered on the hippocampus and amygdala were compromised in male offspring of mothers that experienced social isolation during pregnancy. This pattern of effects suggests that social isolation during pregnancy can have significant impacts on neural networks essential for normal cognitive functions associated with spatial navigation/memory and emotions.

## Supplementary Information

Supplementary Figures.

## Data Availability

The datasets used during the current study are available from the corresponding author upon reasonable request.
