# Supplementary material for: Effects of maternal social isolation on adult rodent offspring cognition
Source: Sci Rep. 2023 May 12;13:7748. doi: 10.1038/s41598-023-34834-0 (PMC10177704; doi:10.1038/s41598-023-34834-0)
Supplement: Supplementary file 1 — Supplementary Figures. [file 41598_2023_34834_MOESM1_ESM.docx]

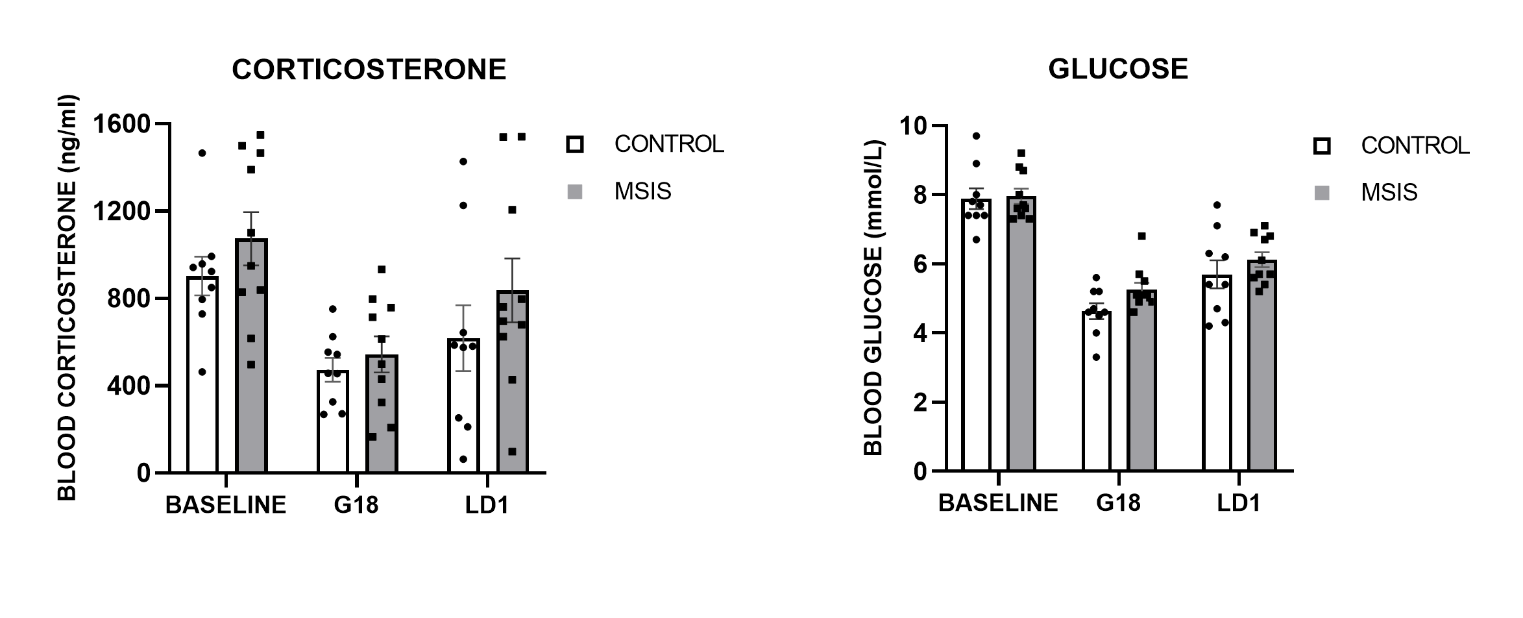


**Supplemental Figure 1.** To ensure the reliability of our dam profile, we included more control (n=9) and maternal social isolation stress (MSIS) (n =10) dams in the analysis. (left) A repeated measures ANOVA on blood corticosterone levels assessed prior to pregnancy (baseline), gestational day 18 (G18) and lactation day 1 (LD1) indicated no significant group [F_(1,17)_ = 1.58, *p* = 0.226], nor group x time point interaction [F_(2,34)_ = 0.34, *p* = 0.72]. A significant effect of time point was found [F_(2,34)_ = 13.8, *p* < 0.001]. Further pairwise comparisons showed a significant decrease in corticosterone levels between baseline and G18, *p <* 0.001*.* (right) A repeated measures ANOVA on blood glucose levels indicated a significant effect of time point [F_(2,34)_ = 106.38, *p* < 0.001], but no significant effects of group nor interaction [F_(1,17)_ = 1.61, *p* = 0.22, F_(2,34)_ = 0.88, *p* = 0.425]. Post-hoc analysis revealed a significant difference in blood glucose levels between baseline to G18 and LD1, *p’*s < 0.001. Further pairwise comparisons revealed a marginally significant group difference at G18, *p* = 0.058, with the MSIS group having elevated blood glucose levels. Data is presented as ± SEM.


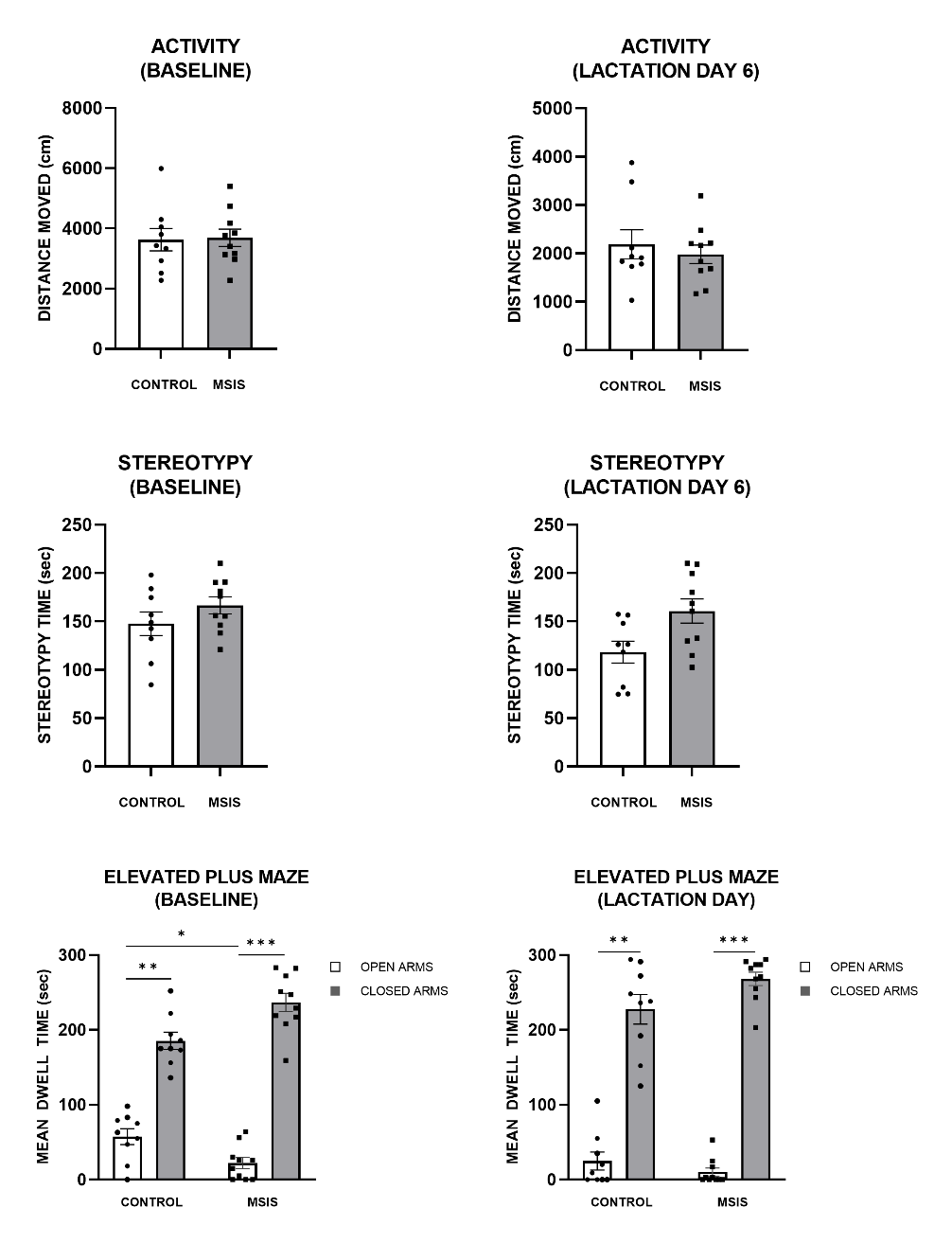


**Supplemental Figure 2.** Additional control and MSIS dams were included to increase group sizes to n = 9, and n = 10, respectively. Univariate ANOVAs performed on activity at baseline and LD6 indicated no group difference, however, activity level for both groups were substantially lower at LD6 (top). Results of the ANOVA on stereotypy time indicated no group difference at baseline or LD6 (middle).

A repeated measures ANOVA performed on the elevated plus maze at baseline showed that there was no difference between the groups [F_(1,17)_ = 3.31, *p* = 0.089] as both groups spent significantly more time in the closed arms than open arms [F_(1,17)_ = 144.35, *p* < 0.001], and a group x dwell arm interaction was also obtained [F_(1,17)_ = 9.237, *p* = .007]. Further Bonferoni post-hoc pairwise comparisons revealed that the MSIS group spent more time in the closed arms than the control group (*p* = 0.012), and spent less time in the open arms than the control group (*p* = 0.007). The results of the elevated plus maze at lactation Day 6 showed that both groups spent more time in the closed vs. open arms [F_(1,17)_ = 192.77, *p* < 0.001], however, this difference was significantly greater in the MSIS than control group [F_(1,17)_ = 6.31, *p* = 0.022]. Data is presented as ± SEM.


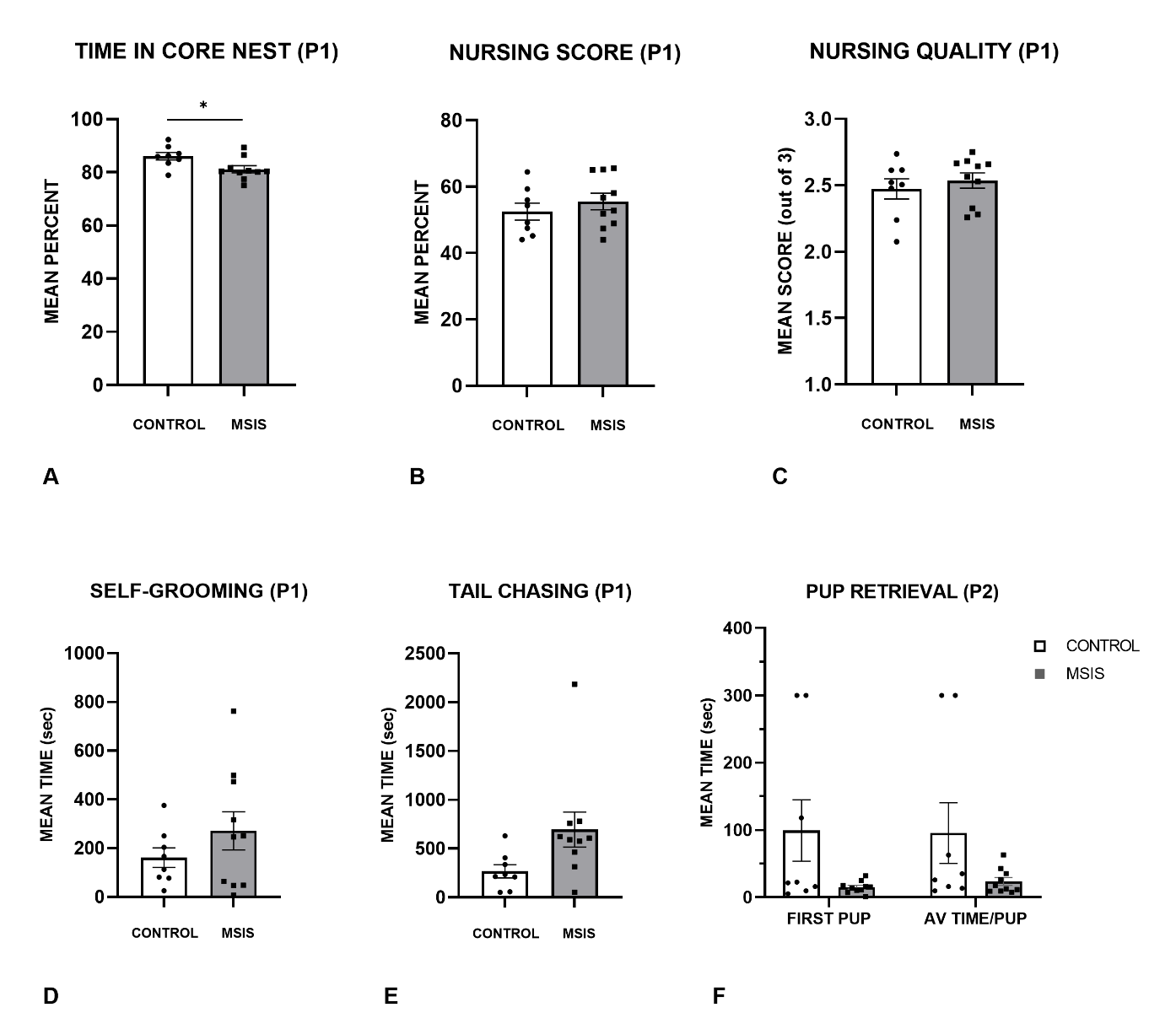


**Supplemental Figure 3.** Additional control and MSIS dams were included to increase group sizes, however, one control dam was removed from the analysis as her behaviours were more than two standard deviations from the other dams, thus the group sizes were n = 8, and n = 10, respectively. Maternal care and behaviour were assessed in control and MSIS dams during the first 10 minutes of every hour over a 24-hr period on post-natal day 1 (P1) and analyzed using univariate Anovas. A significant group effect was found on (A) percent time spent in core nest F_(1,16)_ = 6.52, **p* = 0.021 with the control dams spending more time in the core nest than the MSIS dams. No significant group effect was found on (B) nursing score percentage F_(1,16)_ = 0.72, *p* = 0.41, (C) qualitative nursing score F_(1,16)_ = 0.454, *p* = 0.51, and (D) self-grooming F_(1,16)_ = 1.34, *p* = 0.26. The MSIS group displayed more time tail-chasing (E) than controls and this resulted in a trend towards significance [F_(1,16)_ = 4.14, *p* = 0.059]. (F) There was also a trend for the MSIS dams to retrieve their pups more quickly than control dams (first pup [F_(1,16)_ = 4.08, *p* = 0.054]; average time per pup [F_(1,16)_ = 4.33, *p* = 0.054]). Data is presented as ± SEM.


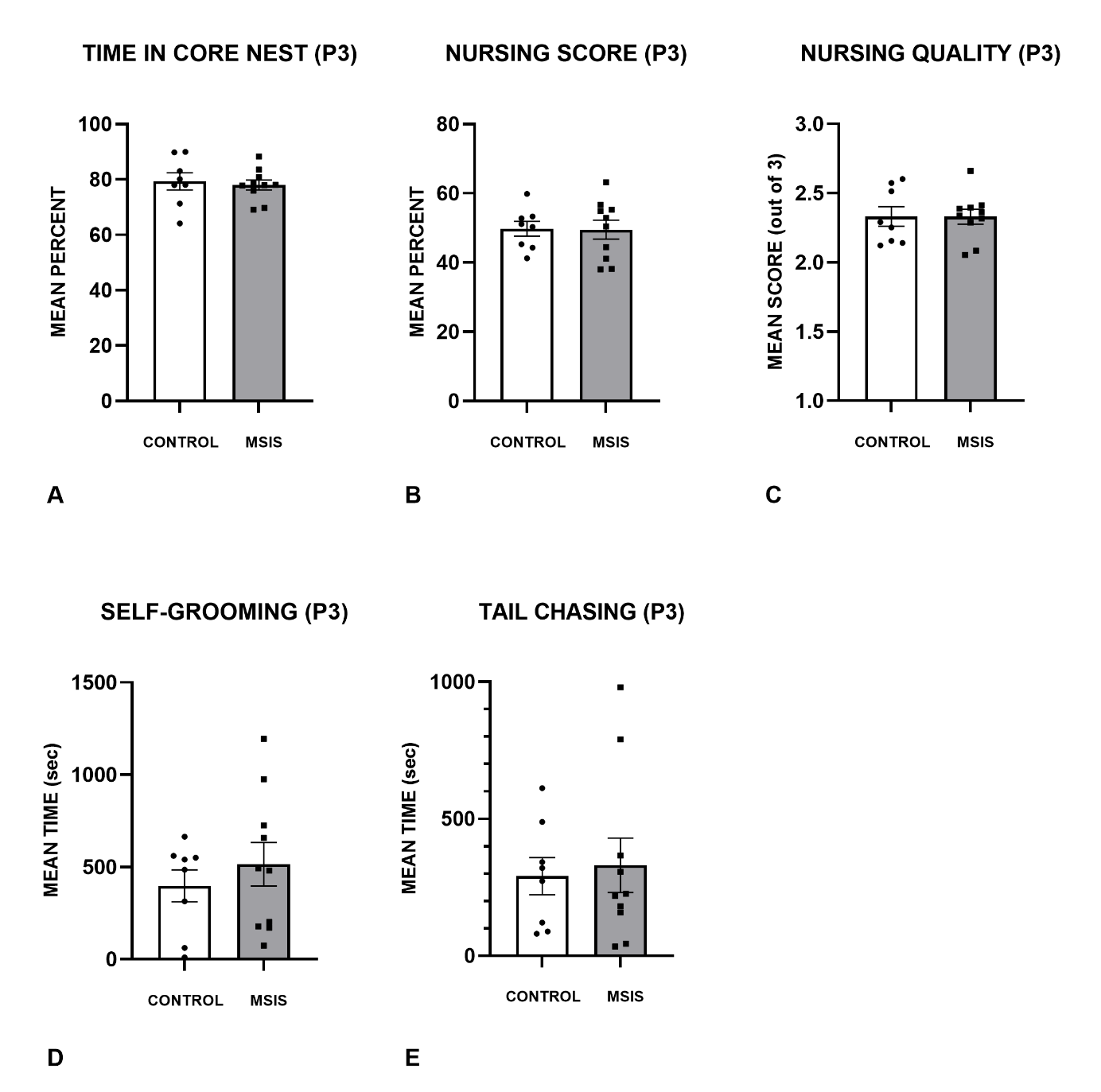


**Supplemental Figure 4.** Additional control and MSIS dams were included to increase group sizes to n = 8, and n = 10, respectively. Maternal care and behaviour were assessed in control and MSIS dams during the first 10 minutes of every hour over a 24-hr period on post-natal day 3 (P3). Univariate Anovas performed on the measures assessed indicated no effect of group on (A) Percent time spent in core nest, (B) percent time spent nursing, (C) qualitative nursing score, (D) self-grooming, and (E) tail-chasing. Thus, there were no differences between the groups on any measure of maternal care nor other behaviors scored. Data is presented as ± SEM.
